# Use of the Behavioral Regulation in Exercise Questionnaire-2 to assess motivation for physical activity in persons with rheumatoid arthritis: an observational study

**DOI:** 10.1007/s00296-021-05079-9

**Published:** 2022-01-09

**Authors:** Vibeke Videm, Mari Hoff, Marthe Halsan Liff

**Affiliations:** 1 Department of Clinical and Molecular Medicine, NTNU – Norwegian University of Science and Technology, Trondheim, Norway; 2 Department of Immunology and Transfusion Medicine, St. Olavs University Hospital, Trondheim, Norway; 3 Department of Neuromedicine and Movement Science, NTNU – Norwegian University of Science and Technology, Trondheim, Norway; 4 Department of Rheumatology, St. Olavs University Hospital, Trondheim, Norway; 5 Department of Rheumatology, Levanger Hospital, Nord-Trøndelag Hospital Trust, Levanger, Norway; 6 Clinic of Orthopedics, Rheumatology and Dermatology, St. Olavs University Hospital, Trondheim, Norway; 7 Department of Clinical and Molecular Medicine, Lab Center 3 East, St. Olavs University Hospital, NO-7006 Trondheim, Norway

**Keywords:** Arthritis, rheumatoid, Motivation, Physical fitness, Exercise test, Physical exercise

## Abstract

Arthritis patients may show little motivation for physical activity (PA), resulting in a sedentary lifestyle. The primary objective of the study was to investigate whether motivation for PA and fulfillment of PA recommendations were associated with cardiorespiratory fitness in patients with RA. The exploratory objective was to study whether university students could be used as controls for RA patients in future studies of PA motivation. Peak oxygen uptake (VO_2peak_) was measured in 93 RA patients. The patients and 354 students filled in the Behavioral Regulation in Exercise Questionnaire-2 (BREQ-2). Data were analyzed using structural equation modeling with adjustment for age and sex. The BREQ-2 scores were also compiled to an overall motivational style “Relative Autonomy Index” as previously published. Mean VO_2peak_ for the RA patients was 32.2 (SD: 9.6) mL × min^−1^ × kg^−1^. Only 29 patients (31%) fulfilled the current recommendations for PA. BREQ-2 scores were associated with measured VO_2peak_ (standardized coefficient 0.33, *p* < 0.001). Whether a person fulfilled the current recommendations for PA was a significant mediator of this effect (standardized coefficients: mediated effect; 0.22, *p* = 0.001, remaining direct effect; 0.11, *p* = 0.18). The Relative Autonomy Index also significantly predicted measured VO_2peak_ (standardized coefficient 0.30, *p* < 0.001). The underlying BREQ-2 factor structure was significantly different between RA patients and university students, and comparison of scores would not be adequate. Motivation for PA was significantly associated with measured VO_2peak_ in RA patients. The effect was mediated by whether the patient fulfilled the current recommendations for PA. Addressing and stimulating motivation is important when intervening to increase PA and cardiovascular fitness in RA patients.

**Supplementary Information:**

The online version contains supplementary material available at 10.1007/s00296-021-05079-9.

## Introduction

Around 0.5–1% of the population suffers from rheumatoid arthritis (RA), a chronic inflammatory disease which may affect any synovial joint, but most often affects the small joints of the hands and feet [1]. Without treatment, joint destruction may ensue over time, so early medical treatment guided by the aim of achieving complete remission has become the standard. Even with treatment, patients often experience pain, fatigue, and reduced quality of life [2].

Previous research has shown that RA patients still have an increased mortality rate compared to the general population [3–6]. Low cardiorespiratory fitness is an important mediator, with direct and indirect effects accounting for 23% of the totally 28% excess relative risk of mortality [6]. Thus, in addition to adequate medication, stimulating RA patients to improve their fitness level should be an important goal in the overall treatment plan [7].

Current guidelines for RA include similar recommendations for aerobic PA as given for the general population by the American College of Sports Medicine (ACSM) and the American Heart Association (AHA): namely moderate-intensity PA ≥ 30 min on ≥ 5 days a week (≥ 150 min per week), high-intensity PA ≥ 20 min ≥ 3 times a week (≥ 75 min per week), or a combination of PA at these intensities [8, 9].

Uptake of exercise recommendations is relatively low in the general population, and several studies show that many RA patients are physically inactive and spend numerous sedentary hours daily [10, 11]. This may have many reasons in addition to physical restrictions, including lack of knowledge about the helpful effects of PA, suitable and accessible exercise programs, and social support [12]. Such barriers may act through effects on motivation to perform PA, and the motivational aspects probably differ between RA patients and the general population.

Motivation comprises a person’s reasons for acting or behaving in a specific way. According to self-determination theory, motivation is a multidimensional concept ranging from intrinsic regulation (completely self-determined behavior regulation) through various intermediate styles (extrinsic regulation, namely behavior performed to obtain an external outcome) to amotivation (no intention to perform a behavior, i.e., completely non-self-determined behavior regulation) [13, 14]. The proposed motivational styles cannot be measured directly, and the instrument Behavioral Regulation in Exercise Questionnaire-2 (BREQ-2) has therefore been developed to enable assessment [14, 15]. The psychological needs for competence in dealing with one’s surroundings, relatedness through satisfying social relationships, and autonomy by doing things from self-determination and not from external control or obligation are proposed as important underlying factors [13]. A substantial body of evidence supports self-determination theory in settings like learning and health-related activities including PA [17].

For studies of motivation for PA in arthritis patients, comparison with a "normal" control group is useful to identify areas where fitness programs would need to differ from those intended for the general population. Previous literature has confirmed good performance of BREQ-2 in diverse groups like students [16, 17], office workers [18], blue-collar workers, white-collar workers and members of a bridge club [19], participants in an Internet-based exercise program [20], and female hospital workers and members of a community church group [21].

We hypothesized that motivation for PA is associated with cardiorespiratory fitness. The gold-standard method to measure cardiorespiratory fitness is using cardiopulmonary exercise testing (CPET) [22]. We further hypothesized that whether the patient fulfills the ACSM/AHA recommendations for PA acts as a mediator between motivation and fitness. The primary aim of the present observational study was therefore to investigate the associations among motivation for PA, fulfillment of PA guidelines, and cardiorespiratory fitness measured using treadmill-based CPET in patients with RA. The secondary aim was to translate the BREQ-2 questionnaire to Norwegian and confirm the psychometric properties of the translated version. We also included an exploratory test of whether university students could be used as controls for RA patients with respect to motivational styles for PA measured using BREQ-2 in future studies. For the exploratory study, we hypothesized that the underlying structure of BREQ-2 would be robust to differences between arthritis patients and students.

## Methods

This was an observational study.

### Participants

For a previously published study regarding cardiorespiratory fitness in RA patients, a convenience sample of 94 individuals performed cardiopulmonary exercise testing (CPET) on a treadmill in 2017–2018 as described below [23]. Diagnosis was confirmed according to the 2010 American College of Rheumatology /European League against Rheumatism 2010 classification criteria [24], and their clinical status was registered. Patients with unstable heart conditions, chronic pulmonary disease necessitating use of oxygen therapy, or physical disability making a treadmill test impossible were excluded. As a pilot test, the participants also filled in a Norwegian version of the BREQ-2 questionnaire, which is further described below. Power calculations were performed for the original study and were therefore not suitable for the present pilot study. The 93 individuals with complete BREQ-2 data were included in the present study. Most patients (*n* = 87) were recruited from the rheumatology outpatient clinic at St. Olavs University Hospital where they either came for regular follow-up visits or participated in a patient-centered follow-up program. Six patients were included after reading a newsletter from the local arthritis association.

For the exploratory aim, students from the Faculty of Medicine and Health Sciences (*n* = 248, Student group 1) and Faculty of Information Technology and Electrical Engineering (*n* = 106, Student group 2) at NTNU—Norwegian University of Science and Technology were included. Students from both faculties were included to achieve a more diverse total control sample. The students were approached before leaving the lecture hall for an intermission and were given oral and written information. Those who wished to participate filled in an anonymous version of the Norwegian BREQ-2 questionnaire, and there were no exclusion criteria.

### Main outcome variable

The main outcome variable was measured cardiorespiratory fitness in the RA patients. Performance of CPET has been described in detail previously [23]. In brief, participants had a 6-min warm-up period, whereafter the walking or running speed of the treadmill (Woodway PPS 55, Waukesha, Wisconsin, USA) was adjusted using an individualized protocol to account for any RA-related physical limitations. The participants were fitted with a facemask (7450 Series V2 CPET mask, Hans Rudolph, Shawnee, Kansas, USA) and a heart rate monitor (H7, Polar Electro, Kempele, Finland), and perceived exertion was rated using the RPE Borg Scale [25]. Gas measurements were recorded every 10 s using a mixing chamber ergospirometry system (Metalyzer II, Cortex Biophysik Gmbh, Leipzig, Germany) and workload was increased gradually until exhaustion. The following criteria were used to define maximal oxygen uptake (VO_2max_): (1) VO_2_ leveling off (< 2 mL × min^−1^ × kg^−1^) despite increase in workload and (2) respiratory exchange ratio ≥ 1.05. If these criteria were not met, the participant’s VO_2peak_ was determined, defined as the mean of the three successive highest VO_2_ registrations achieved during the CPET. The term VO_2peak_ is used for all patients for simplicity even if 83% qualified for VO_2max_.

### Study factors

The main study factor was the assessed motivation for physical activity as measured by the BREQ-2 questionnaire [15]. The 19 items of the English version of the BREQ-2 questionnaire were translated to Norwegian by a bilingual person and back-translated to English by another bilingual person (Online Resource 1—Norwegian version of the BREQ-2 questionnaire). A style of Norwegian close to everyday language was chosen as opposed to more formal written language. The motivational styles are denoted as (1) intrinsic regulation, where PA is performed because the person finds it enjoyable in itself (for example, “I enjoy my exercise sessions”), (2) identified regulation, where PA may help a person achieve his or her goals (for example, “I value the benefits of exercise”), (3) introjected regulation, where PA is performed to increase one’s self-esteem or avoid negative feelings (for example, “I feel ashamed when I miss an exercise session”), (4) external regulation, where PA is performed due to rewards or punishments given by someone else (for example, “I feel under pressure from my friends/family to exercise"), and (5) amotivation, where the person has no intention to perform PA (for example, “I think exercising is a waste of time”) [15, 18, 26]. The complete list of items is given in Table 1.

**Table 1:** BREQ-2 items and scores

BREQ-2 factor	BREQ-2 items^a^	Persons with rheumatoid arthritis (*n* = 93)	Student group 1 (*n* = 248)	Student group 2 (*n* = 106)
Intrinsic regulation		3.91 (0.90)	4.02 (0.87)	4.05 (0.80)
	4. I exercise because it’s fun			
	10. I enjoy my exercise sessions			
	15. I find exercise a pleasurable activity			
	18. I get pleasure and satisfaction from participating in exercise			
Identified regulation		3.91 (0.78)	4.04 (0.82)	4.02 (0.89)
	3. I value the benefits of exercise			
	8. It’s important to me to exercise regularly			
	14. I think it is important to make the effort to exercise regularly			
	17. I get restless if I don’t exercise regularly			
Introjected regulation		2.36 (0.81)	2.77 (0.98)	2.78 (1.06)
	2. I feel guilty when I don’t exercise			
	7. I feel ashamed when I miss an exercise session			
	13. I feel like a failure when I haven’t exercised in a while			
External regulation		1.34 (0.57)	1.60 (0.68)	1.69 (0.63)
	1. I exercise because other people say I should			
	6. I take part in exercise because my friends/family/partner say I should			
	11. I exercise because others will not be pleased with me if I don’t			
	16. I feel under pressure from my friends/family to exercise			
Amotivation		1.19 (0.46)	1.15 (0.37)	1.20 (0.45)
	5. I don’t see why I should exercise			
	9. I can’t see why I should bother exercising			
	12. I don’t see the point in exercising			
	19. I think exercising is a waste of time			
Relative autonomy index	NA	10.9 (5.4)	10.7 (5.4)	10.4 (4.8)

*BREQ*-*2* Behavioral Regulation in Exercise Questionnaire-2, *NA* not applicable

^a^[15]

The BREQ-2 items have 5-point Likert scales ranging from 0 (“not true for me”) to 4 (“very true for me”). The mean score of the three to four items related to each factor was calculated, giving the individual’s score for each of the five types of motivation for exercise [15]. A relative autonomy index (RAI) was also calculated where the score for each of the five factors was weighted, and the resulting numbers were summed. The weightings are: Intrinsic regulation: 3, identified regulation: 2, introjected regulation: − 1, external regulation: − 2, amotivation: − 3. The possible RAI values range from − 24 to 20 with higher scores indicating more self-determined motivation [26].

### Other variables

The RA patients provided information about the frequency, duration, and intensity of the physical activity they usually perform, which was used to evaluate whether they fulfilled the ACSM/AHA recommendations for PA [8]. For descriptive purposes, RA disease activity was measured by Disease Activity Score 20 (DAS28) including the high-sensitivity C-reactive protein concentration [27]. Assessment of functional status was measured by the modified Health Assessment Questionnaire (mHAQ), which includes eight questions regarding the ability to perform common daily activities during the last week with answers given on a 4-point Likert scale [29]. Use of disease-modifying antirheumatic drugs was registered.

In addition to the BREQ-2 questionnaire, the included students also provided information about their sex and age, but not other demographical or social data.

### Procedures

The study was performed in compliance with the Helsinki Declaration and was approved by the Regional Committee for Medical and Health Research Ethics (#20734). All participants gave informed consent.

### Statistical analysis

Data are given as frequency (percentage) or mean (standard deviation), and descriptive statistics were compared among groups using the Chi-square test or *T* test. *p* values < 0.05 were considered significant.

In the RA patients, associations between motivation, fulfillment of the ACSM/AHA guidelines for PA and VO_2peak_ as outcome were analyzed using Structural Equation Modeling (SEM) [30]. For these analyses, *n* = 92 due to missing questionnaire data on performed PA for one person. In SEM, a model showing the proposed relationships among the variables is drawn, and the method then tests whether the observed data fit with the model, using a series of common fit indices (Online Resource 2—Common fit indices for structural equation models) [30]: the Chi-square test, root mean squared error of approximation (RMSEA), Tucker Lewis index (TLI), comparative fit index (CFI), and standardized root mean square residual (SRMR). In a standardized model, all variables are measured in the same unit, namely standard deviations. This makes the coefficient sizes directly comparable, which would not be so when for example BREQ-2 scores are measured in numbers from 0 to 4 and age in years. Due to non-normal distributions, standard errors were calculated using the Satorra–Bentler method [15].

**Fig. 1:**
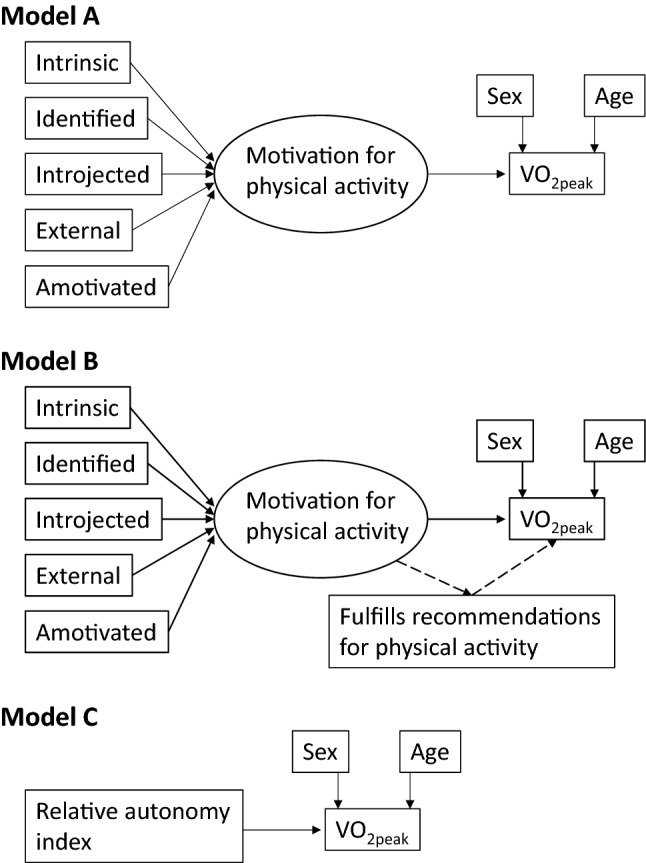
Models for associations of motivation for physical activity and cardiorespiratory fitness. Model A assumes that the BREQ-2 factors together define a latent factor Motivation for physical activity, which in turn predicts the measured VO_2peak_. Model B assumes that whether the person fulfills the ACSM/AHA recommendations for physical activity (yes/no) is a mediator for the effect of this predictive latent factor. Model C assumes that the calculated relative autonomy index from the BREQ-2 factors may substitute for the individual BREQ-2 factors and the latent Motivation factor. All models were adjusted for sex (0 = woman, 1 = man) and age (years). Coefficients were standardized and their size is therefore directly comparable. *ACSM* American College of Sports Medicine, *AHA* American Heart Association, *BREQ-2* Behavioral Regulation in Exercise Questionnaire-2, *VO*_*2peak*_ peak oxygen uptake

The proposed models for the primary aim are shown in Fig. 1. The first model proposed that the five BREQ-2 factors were measurements of a latent variable Motivation for PA, which in turn was associated with the measured VO_2peak_. The second model was similar, but further proposed that whether the person fulfilled the ACSM/AHA recommendations for PA (yes/no) acted as a mediator between Motivation for PA and the measured VO_2peak_. Because analysis in a real-world setting would be easier using RAI instead of the five separate BREQ-2 factors, a third model proposed that the calculated RAI was associated with the measured VO_2peak_, thus not assuming any latent Motivation for PA factor. All models included adjustment for sex and age, which are known to influence VO_2peak_ [11]. Model fit was assessed using the fit indices mentioned above. The sample size was small for SEM, so a sensitivity analysis was performed where the main models were run in bootstrap samples (*n* = 300) for bias correction of coefficients and standard errors.

For the secondary aim, namely, to test whether the original BREQ-2 5-factor structure fit well in our data using the translated items, confirmatory factor analysis by means of SEM was employed [29]. Based on item correlations, they are reduced into a set of underlying latent factors, in this case those indicated in the original BREQ-2 publication [15]. The item correlations with these factors are denoted their factor loadings. Model fit was assessed using the fit indices mentioned above. Different models were compared using the likelihood ratio (LR) test. Internal consistency (reliability) of the factors was measured using Cronbach’s alpha, where values > 0.7 are considered good [30].

Details regarding the SEM methods used in the exploratory study to evaluate whether the five BREQ-2 factors could be compared between students and RA patients are given in Online Resource 3—Supplementary methods—Comparison of BREQ-2 factor structure between RA patients and students.

Statistical analysis was performed using Stata/MP (v.16.1, StataCorp, College Station, TX, USA).

## Results

Table 2 shows the participant characteristics. Most of the patients were seropositive and had long-standing RA. As expected, the RA patients were significantly older than the students (*p* < 0.001) (Online Resource 4—Participant characteristics in exploratory study). Only 29 patients (31%) fulfilled the ACSM/AHA recommendations for PA. BREQ-2 results are given in Table 1. Both RA patients and students scored highest on intrinsic and identified regulation and low on amotivation. Mean RAI was between 10 and 11 for both groups.

**Table 2:** Participant characteristics

Variable	Persons with rheumatoid arthritis (*n* = 93)
Women	68 (73%)
Age (years)	59 (12)
Height (m)	1.69 (0.90)
Weight (kg)	76.4 (12.3)
Smoking	
Never smoked	35 (38%)
Previous smoker	51 (55%)
Present smoker	7 (8%)
Fulfills ACSM/AHA recommendations	29 (31%)
Disease duration (years)	12 (9)
Seropositive (anti-citrullinated peptide antibody and/or rheumatoid factor positive)	75 (81%)
Uses conventional DMARD	74 (80%)
Uses biological DMARD	54 (58%)
DAS28	2.56 (1.04)
mHAQ	0.26 (0.31)
Peak oxygen uptake (mL × min^−1^ × kg^−1^)	32.2 (9.6)

Data are given as number (percent) or mean (SD). Some of the data for persons with rheumatoid arthritis have been published previously [23]

*ACSM* American College of Sports Medicine, *AHA* American Heart Association, *DAS28* disease activity score including high-sensitivity C-reactive protein concentration, *DMARD* disease-modifying antirheumatic drug, *mHAQ* modified Health Assessment Questionnaire

For the main aim regarding motivation and VO_2peak_ in the RA patients, Model A (Fig. 1) had good fit and was therefore compatible with the hypothesis that a latent factor Motivation for PA based on the five BREQ-2 factors was associated with the measured VO_2peak_ (Chi square 15.18, df = 15, *p* = 0.44, RMSEA = 0.01, TLI = 1.00, CFI = 1.00, SRMR = 0.06). The associations with Motivation for PA were positive and significant for intrinsic and identified regulation, non-significant for introjected regulation, and negative and significant for external motivation and amotivation (Table 3).

**Table 3:** Analysis of associations with cardiorespiratory fitness

	Associations with latent factor Motivation for PA^a^ Coefficient (*p* value)		Associations with VO_2peak_ Coefficient (*p* value)	
Model A	Intrinsic regulation	0.92 (*p* < 0.001)	Motivation for PA	0.33 (*p* < 0.001)
	Identified regulation	0.78 (*p* < 0.001)	Sex^b^	0.51 (*p* < 0.001)
	Introjected regulation	0.08 (*p* = 0.49)	Age	− 0.62 (*p* < 0.001)
	External regulation	− 0.31 (*p* = 0.01)		
	Amotivation	− 0.60 (*p* < 0.001)		
Model B	Intrinsic regulation	0.89 (*p* < 0.001)	Motivation for PA	
			Direct effect	0.11 (*p* = 0.18)
			Indirect effect	0.22 (*p* = 0.001)
	Identified regulation	0.81 (*p* < 0.001)	Sex	0.52 (*p* < 0.001)
	Introjected regulation	0.09 (*p* = 0.46)	Age	− 0.61 (*p* < 0.001)
	External regulation	− 0.33 (*p* = 0.01)		
	Amotivation	− 0.61 (*p* < 0.001)		
Model C			Relative autonomy index	0.54 (*p* < 0.001)
			Sex	0.51 (*p* < 0.001)
			Age	− 0.63 (*p* < 0.001)

*BREQ-2* Behavioral Regulation in Exercise Questionnaire-2, *PA* physical activity, *RA* rheumatoid arthritis

^a^The latent factor Motivation for PA is illustrated in Fig. 1

^b^Sex: 0 = female, 1 = male

Model B (Fig. 1, Table 3) showed that whether a person fulfills the recommendations for PA was a significant mediator of the association of Motivation for PA with VO_2peak_, whereas the direct effect became non-significant. There were minimal changes for the associations of the BREQ-2 factors with motivation for PA. Model B had good fit (Chi square 22.77, df = 21, *p* = 0.30, RMSEA = 0.026, TLI = 0.99, CFI = 0.99, SRMR = 0.06).

In Model C (Fig. 1, Table 3), RAI was significantly positively associated with VO_2peak_. The coefficient (0.30) was close to that of Motivation for PA (0.33) in Model A. All variables were treated as observed in this saturated model, which had excellent fit (Chi-square test: not applicable, RMSEA *p* = 1.00, TLI = 1.00, CFI = 1.00, SRMR = 0.00). The coefficients for the adjustment variables sex and age were essentially equal in all three models (Table 3), confirming the well-known findings of higher VO_2peak_ in men and lower VO_2peak_ with older age.

The sensitivity analysis in bootstrap samples confirmed that the models had little bias despite the sample size, with small differences in coefficients or CI (data not shown).

For the secondary aim of testing the psychometric properties of the original BREQ-2 factors in our population, all items correlated with their proposed latent factors and the overall model fit was good (Chi square 304.27, df = 136, *p* < 0.01, RMSEA = 0.053, CFI = 0.94, TLI = 0.92, SRMS = 0.057) (Online Resource 5—BREQ-2 psychometric properties). A significant Chi-square test is common in large studies [30] and has been found in other studies of BREQ-2 [20]. The factors were partly correlated in accordance with self-determination theory that the regulatory styles form a continuum from intrinsic regulation to amotivation, and with strongest correlation between factors more closely related [15]. Because the correlation between intrinsic and identified regulation was very high (0.93), we tested a model where the items belonging to these factors were pooled as one factor. The fit for this 4-factor model was significantly worse than the original 5-factor model (LR test Chi square 96.56, df = 4, *p* < 0.001). Internal consistency (reliability) of the five factors was good (Cronbach’s alphas from 0.73 to 0.87) (Online Resource 5—BREQ-2 psychometric properties).

In the exploratory study, the results showed that using the students as a control population for the RA patients would not be adequate, as the factor structure of BREQ-2 was significantly different in these two populations. Further details are given in Online Resource 6 (LR tests for measurement invariance between students and patients with RA).

## Discussion

The study confirmed that the latent factor Motivation for PA was significantly associated with measured VO_2peak_ in RA patients. Furthermore, whether the patient fulfilled the ACSM/AHA recommendations for PA mediated the effect of Motivation for PA on VO_2peak_. The study also showed that the RAI could act as useful summary measure for BREQ-2, with a similar effect size as Motivation for PA, and with stable coefficients for the adjustment variables age and sex. The original 5-factor BREQ-2 model had retained its psychometric properties following translation into everyday Norwegian. However, despite overall good fit, the factor structure was significantly different between RA patients and university students, indicating that choice of control group for BREQ-2 scores in arthritis patients is not trivial.

We have previously shown that the average fitness level is lower in RA patients and declines more rapidly with age than in healthy individuals of the same age [11]. The present study points at motivation as a key factor to address when promoting PA for RA patients, which has important clinical implications. The results also contribute to strengthening the evidence for the recent EULAR (European Alliance of Associations for Rheumatology) recommendations, which focus on the need to base interventions on individual aims and consider barriers and facilitators [9]. In a recent randomized controlled trial addressing PA motivation in RA patients, training was equal for both groups, and a self-determination theory-based psychological intervention resulted in higher autonomous motivation [31]. In turn, autonomous motivation predicted self-reported PA and subjective vitality after 3 months. These results support our present findings and demonstrate that motivation may be modified using appropriate methods. However, self-reported PA may be biased. A strength of our study is that we used CPET to quantify VO_2peak_ instead of self-reported PA or indirect measurements to quantify of fitness [32].

Our study also showed that it is plausible to assume that a person's different forms of motivational regulation for PA come together as an overall underlying factor, which influences the performed activities that in turn may modify measured fitness. The direction of the association was positive as expected, indicating that patients with higher intrinsic motivation had higher VO_2peak_. The interpretation was strengthened by the finding that the effect of motivation was mediated through fulfillment or not of widely accepted recommendations for a clinically relevant level of PA, which take both frequency, duration, and intensity into consideration. These aspects of PA are important, as high-intensity activities more efficiently increase fitness and there is a PA dose effect as well [33]. Our study may also be considered an external validation of the BREQ-2 questionnaire by demonstrating an association of the motivational styles with a relevant outcome, i.e., VO_2peak_.

The present study also confirmed that the summary measure RAI may be useful in studies where the detailed motivational regulation of PA is of less importance. Using the RAI leads to loss of detail and the proposed weighting may not be correct, but statistical analysis becomes easier when latent variables are not included. Several published studies have used the RAI for this reason [19, 26, 27, 32], but to our knowledge, the validity of RAI has not previously been confirmed based on CPET results. Filling in the BREQ-2 questionnaire only takes a few minutes and use of the RAI may be a simple way to evaluate motivation for PA in future studies of patients with inflammatory arthritis.

The exploratory part of our study has important consequences regarding the choice of control population for future studies of motivation for PA in arthritis patients. We used university students because a sufficiently large number of participants could easily be recruited, but also as a "stress test" because they were different in age from most of the RA patients. Showing a similar factor structure in these two diverse groups would have simplified selection of controls in future studies. Our findings are somewhat different from an investigation of participants in an Internet-based exercise program, where there were no differences between the 18–45 years and 47–78 years age groups or between men and women [20].

The study has some limitations. There may be a selection bias regarding which RA patients who sign up for a CPET study. However, the VO_2peak_ measurements covered a wide range and only 1/3 of the patients fulfilled the current PA recommendations, which argues against a strong selection bias toward the more fit persons. The sample of RA patients was small for SEM and too small for more detailed analysis, including of the potential influence of disease-modifying antirheumatic drugs or disease activity. However, the sensitivity analysis indicated little bias. A weakness using SEM is that other models may fit the data equally well or better, and that only associations are measured, not causation.

In conclusion, the study demonstrated that motivation for PA was significantly associated with measured VO_2peak_ in RA patients. The effect was mediated by whether the patient fulfilled the current recommendations for PA. The results should be confirmed in a larger future study, which would also permit more detailed analysis. Addressing and stimulating motivation is important when intervening to increase PA and cardiorespiratory fitness in RA patients, and further studies using interventions aimed at strengthening the intrinsic motivation for PA in RA patients are needed.

## Supplementary Information

Below is the link to the electronic supplementary material.

Supplementary file1 (PDF 127 KB)

Supplementary file2 (PDF 108 KB)

Supplementary file3 (PDF 105 KB)

Supplementary file4 (PDF 116 KB)

Supplementary file5 (PDF 145 KB)

Supplementary file6 (PDF 110 KB)

## Data Availability

No additional data are available.
