# Supplementary material for: Use of the Behavioral Regulation in Exercise Questionnaire-2 to assess motivation for physical activity in persons with rheumatoid arthritis: an observational study
Source: Rheumatol Int. 2022 Jan 9;42(11):2039–47. doi: 10.1007/s00296-021-05079-9 (PMC9510116; doi:10.1007/s00296-021-05079-9)
Supplement: Supplementary file 1 — Supplementary file1 (PDF 127 KB) [file 296_2021_5079_MOESM1_ESM.pdf]

Videm V, Hoff M, Liff MH: Use of the Behavioral Regulation in Exercise Questionnaire-2 to assess motivation for physical activity in persons with rheumatoid arthritis – an observational study
Rheumatology International
Corresponding author: Vibeke Videm, Department of Clinical and Molecular Medicine, NTNU - Norwegian University of Science and Technology and Department og Immunology and Transfusion Medicine, St. Olavs University Hospital, Trondheim, Norway. E-mail: vibeke.videm@ntnu.no

Online Resource 1: Norwegian version of the BREQ-2 questionnaire[a]

BREQ-2: Grunner til å trene eller være fysisk aktiv
Vi ønsker å undersøke hvorfor personer deltar eller ikke deltar i fysisk aktivitet og trening. Vennligst merk av i hvilken grad påstandene under passer for deg ved å sette kryss i én av rutene på skalaen på hver linje. Det finnes ingen riktige eller gale svar – vi ønsker bare å få en beskrivelse av dine personlige følelser rundt trening. Vennligst svar på alle spørsmålene.

	Ikke sant for meg		Delvis sant for meg		Veldig sant for meg
1. Jeg trener fordi andre sier at jeg bør					
2. Jeg får dårlig samvittighet når jeg ikke trener					
3. Jeg verdsetter fordelene ved trening					
4. Jeg trener fordi det er gøy					
5. Jeg skjønner ikke hvorfor jeg skulle være nødt til å trene					
6. Jeg deltar i trening fordi venner/partner/familie mener jeg bør					
7. Jeg skammer meg når jeg går glipp av en treningsøkt					
8. Det er viktig for meg å trene regelmessig					
9. Jeg skjønner ikke hvorfor jeg skulle gidde å trene					
10. Jeg liker treningsøktene mine					
11. Jeg trener fordi andre ikke ville være fornøyde med meg om jeg lar være					
12. Jeg ser ikke noe poeng i å trene					
13. Jeg føler meg mislykket når jeg ikke har trent på en stund					
14. Jeg mener det er viktig å gjøre en innsats for å trene regelmessig					
15. Jeg synes trening er en lystbetont aktivitet					
16. Jeg føler press fra venner/familie om å trene					
17. Jeg blir rastløs om jeg ikke trener regelmessig					
18. Jeg blir glad og tilfreds av å delta i trening					
19. Jeg synes trening er bortkastet tid					

[a]Translated from: Markland D, Tobin V. A modification to the Behavioural Regulation in Exercise Questionnaire to include an assessment of amotivation. J Sport Exerc Psychol 2004;26:191-196.
