# Supplementary material for: Use of the Behavioral Regulation in Exercise Questionnaire-2 to assess motivation for physical activity in persons with rheumatoid arthritis: an observational study
Source: Rheumatol Int. 2022 Jan 9;42(11):2039–47. doi: 10.1007/s00296-021-05079-9 (PMC9510116; doi:10.1007/s00296-021-05079-9)
Supplement: Supplementary file 2 — Supplementary file2 (PDF 108 KB) [file 296_2021_5079_MOESM2_ESM.pdf]

Videm V, Hoff M, Liff MH: Use of the Behavioral Regulation in Exercise Questionnaire-2 to assess motivation for physical activity in persons with rheumatoid arthritis – an observational study
Rheumatology International
Corresponding author: Vibeke Videm, Department of Clinical and Molecular Medicine, NTNU - Norwegian University of Science and Technology and Department og Immunology and Transfusion Medicine, St. Olavs University Hospital, Trondheim, Norway. E-mail: vibeke.videm@ntnu.no

Online Resource 2: Common fit indices for structural equation models[a,b]

Fit index	Abbreviation	Explanation	Indication of fit
Chi square test		Overall fit compared to model with no correlations	p>0.05: good fit, but too sensitive in large studies
Root Mean Squared Error of Approximation	RMSEA	Compares observed covariance matrix and model covariance matrix,<br>adjusts for model complexity and sample size	p=0.05-0.08: good fit<br>p<0.05: excellent fit
Tucker Lewis Index	TLI	Compares model to model with no correlations, adjusts for model complexity	≥0.90: good fit<br>>0.95: excellent fit
Comparative Fit Index	CLI	Compares model to model with no correlations, adjusts for model degrees of freedom	≥0.90: good fit<br>>0.95: excellent fit
Standardized Root Mean Square Residual	SRMR	Evaluates residuals between observed data and model	≤0.10: good fit

[a] Mehmetoglu M, Jakobsen TG (2017) Applied statistics using Stata. A guide for the social sciences. Sage Publications, pp 305-309
[b] Baldwin SA (2019) Psychological statistics and psychometrics using Stata. Stata Press, pp 392-395
