# Supplementary material for: Use of the Behavioral Regulation in Exercise Questionnaire-2 to assess motivation for physical activity in persons with rheumatoid arthritis: an observational study
Source: Rheumatol Int. 2022 Jan 9;42(11):2039–47. doi: 10.1007/s00296-021-05079-9 (PMC9510116; doi:10.1007/s00296-021-05079-9)
Supplement: Supplementary file 3 — Supplementary file3 (PDF 105 KB) [file 296_2021_5079_MOESM3_ESM.pdf]

Videm V, Hoff M, Liff MH: Use of the Behavioral Regulation in Exercise Questionnaire-2 to assess motivation for physical activity in persons with rheumatoid arthritis – an observational study
Rheumatology International
Corresponding author: Vibeke Videm, Department of Clinical and Molecular Medicine, NTNU - Norwegian University of Science and Technology and Department og Immunology and Transfusion Medicine, St. Olavs University Hospital, Trondheim, Norway. E-mail: vibeke.videm@ntnu.no

Online Resource 3: Supplementary methods - Comparison of BREQ-2 factor structure between RA patients and students

For comparison of motivational styles for exercise in RA patients and controls using the BREQ-2 questionnaire, it is important that the underlying factor structure is similar in the two groups. If not, structural differences could be mistaken for intergroup differences.

To investigate compare the underlying BREQ-2 factor structure between RA patients and controls, several forms of measurement invariance were studied [1]: metric invariance (whether the items load equally onto their factors in the groups), scalar invariance (whether one group consistently scores higher or lower on the factors when the latent structure is accounted for, i.e., if the responses are differently calibrated), and residual invariance (whether the differences between the model and the observed data have equal variances in the groups, i.e., whether the precision of the measurements is similar). Without metric and scalar invariance inter-group comparisons do not make sense because the instrument works differently between the groups. When metric and scalar invariance are present but not residual invariance, intergroup comparisons are valid, but interpretation is more difficult due to differences in precision.

Measurement invariance for each factor was evaluated using a hierarchy of Structural Equation Modeling (SEM) models with different constraints, where model fit between pairs of models by the likelihood ratio (LF) test would not be significantly different if the relevant form of measurement invariance between the groups was present [1]. For example, if a model that constrains the item loadings to be equal between the groups fits equally well as a model without this constraint, there is metric invariance. If the constrained model has worse fit, the observed data do not agree with the assumption of metric invariance, and further assessment of scalar and residual invariance is not relevant [1].

1. Baldwin SA (2019) Psychological statistics and psychometrics using Stata. Stata Press, pp 392-395
