# Supplementary material for: Use of the Behavioral Regulation in Exercise Questionnaire-2 to assess motivation for physical activity in persons with rheumatoid arthritis: an observational study
Source: Rheumatol Int. 2022 Jan 9;42(11):2039–47. doi: 10.1007/s00296-021-05079-9 (PMC9510116; doi:10.1007/s00296-021-05079-9)
Supplement: Supplementary file 4 — Supplementary file4 (PDF 116 KB) [file 296_2021_5079_MOESM4_ESM.pdf]

Videm V, Hoff M, Liff MH: Use of the Behavioral Regulation in Exercise Questionnaire-2 to assess motivation for physical activity in persons with rheumatoid arthritis – an observational study
Rheumatology International
Corresponding author: Vibeke Videm, Department of Clinical and Molecular Medicine, NTNU - Norwegian University of Science and Technology and Department og Immunology and Transfusion Medicine, St. Olavs University Hospital, Trondheim, Norway. E-mail: vibeke.videm@ntnu.no

Online Resource 4: Participant characteristics in exploratory study

Variable	Persons with rheumatoid arthritis (n=93)	Student group 1 (n=248)	Student group 2 (n=106)	P-value[a]
Women	68 (73%)	156 (63%)	30 (28%)	<0.001
Age (years)	59 (12)	23 (3)	21 (1)	<0.001

[a]Chi-square test or ANOVA
