# Supplementary material for: Use of the Behavioral Regulation in Exercise Questionnaire-2 to assess motivation for physical activity in persons with rheumatoid arthritis: an observational study
Source: Rheumatol Int. 2022 Jan 9;42(11):2039–47. doi: 10.1007/s00296-021-05079-9 (PMC9510116; doi:10.1007/s00296-021-05079-9)
Supplement: Supplementary file 5 — Supplementary file5 (PDF 145 KB) [file 296_2021_5079_MOESM5_ESM.pdf]

Videm V, Hoff M, Liff MH: Use of the Behavioral Regulation in Exercise Questionnaire-2 to assess motivation for physical activity in persons with rheumatoid arthritis – an observational study
Rheumatology International
Corresponding author: Vibeke Videm, Department of Clinical and Molecular Medicine, NTNU - Norwegian University of Science and Technology and Department og Immunology and Transfusion Medicine, St. Olavs University Hospital, Trondheim, Norway. E-mail: vibeke.videm@ntnu.no

Online Resource 5: BREQ-2 psychometric properties

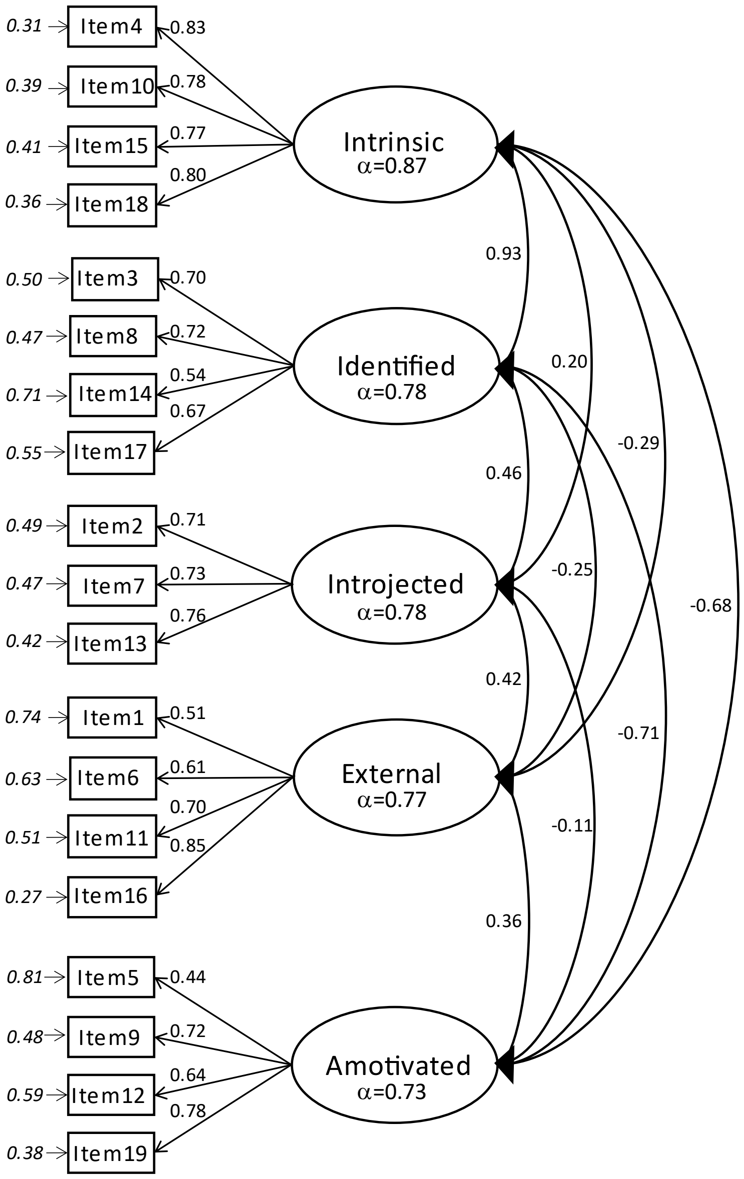

Results from confirmatory factor analysis (n=447): Numbers indicate correlations between items and their corresponding factors, and among factors. Left-hand numbers in italics show item error variances. Cronbach's alpha (α) for each factor indicates internal consistency (reliability).

Abbreviation: BREQ-2: Behavioral Regulation in Exercise Questionnaire-2
