# Supplementary material for: Use of the Behavioral Regulation in Exercise Questionnaire-2 to assess motivation for physical activity in persons with rheumatoid arthritis: an observational study
Source: Rheumatol Int. 2022 Jan 9;42(11):2039–47. doi: 10.1007/s00296-021-05079-9 (PMC9510116; doi:10.1007/s00296-021-05079-9)
Supplement: Supplementary file 6 — Supplementary file6 (PDF 110 KB) [file 296_2021_5079_MOESM6_ESM.pdf]

Videm V, Hoff M, Liff MH: Use of the Behavioral Regulation in Exercise Questionnaire-2 to assess motivation for physical activity in persons with rheumatoid arthritis – an observational study
Rheumatology International
Corresponding author: Vibeke Videm, Department of Clinical and Molecular Medicine, NTNU - Norwegian University of Science and Technology and Department og Immunology and Transfusion Medicine, St. Olavs University Hospital, Trondheim, Norway. E-mail: vibeke.videm@ntnu.no

Online Resource 6: Likelihood ratio tests for measurement invariance between students and patients with rheumatoid arthritis (RA)

Factor	Metric invariance	Scalar invariance
Intrinsic regulation	Chi-square 2.75 (df=3) p=0.43	Chi-square 22.7 (df=3) p<0.001
Identified regulation	Chi-square 3.53 (df=3) p=0.34	Chi-square 46.09 (df=3) p<0.001
Introjected regulation	Chi-square 1.17 (df=2) p=0.56	Chi-square 9.07 (df=2) p=0.01
External regulation	Chi-square 10.73 (df=3) p=0.01	NA
Amotivation	Chi-square 16.93 (df=3) p<0.001	NA

This table shows results from tests for measurement invariance for each BREQ-2 factor between RA patients and students. Intrinsic, identified, and introjected regulation showed metric invariance (non-significant Chi-square test), but not scalar invariance (significant Chi-square test). Thus, residual variance was not relevant to test for these factors. External regulation and amotivation did not show metric invariance and further testing was not relevant. Taken together, the results showed that using the students as a control population for the RA patients would not be adequate, as the factor structure of BREQ-2 was significantly different in these two populations.

Abbreviations: df: degrees of freedom; NA: not applicable
